# Experiences of Discrimination and Alcohol Involvement Among Young Adults at the Intersection of Race/Ethnicity and Gender

**DOI:** 10.1007/s40615-024-02191-x

**Published:** 2024-10-07

**Authors:** Hector Ismael Lopez-Vergara, William Rozum, Jodi M. Sutherland Charvis, Sydney Iacoi, Chrystal Vergara-Lopez, L. A. R. Stein

**Affiliations:** 1 Department of Psychology, The University of Rhode Island, 306 Chafee Hall, 142 Flagg Road Kingston, South Kingstown, RI 02881 USA; 2 Department of Psychiatry and Human Behavior, The Warren Alpert Medical School of Brown University, Providence, RI USA; 3 Center for Behavioral and Preventive Medicine, The Miriam Hospital, Providence, RI USA; 4 Department of Behavioral & Social Sciences and The Center for Alcohol & Addiction Studies, Brown University School of Public Health, Providence, RI USA; 5 Department of Behavioral Healthcare, Developmental Disabilities & Hospitals, Cranston, RI USA

**Keywords:** Discrimination, Alcohol, Gender, Race, Ethnicity, Measurement invariance, Young adults

## Abstract

Although discrimination is an important social determinant of alcohol involvement, there is a dearth of research testing these associations across race/ethnicity and gender. This is an important research gap given that experiences of discrimination and therefore links with alcohol involvement may vary as a function race/ethnicity and gender intersectional identities. We tested for measurement invariance in discrimination and alcohol involvement and examined group differences in means and covariances. The sample consisted of *n* = 1187 young adults (ages 18–26; *n* = 193 Black women, *n* = 209 Latina women, *n* = 186 White women, *n* = 198 Black men, *n* = 203 Latino men, and *n* = 198 White men). We found evidence for differential item functioning for discrimination and alcohol involvement that violated assumptions needed to make manifest between-group comparisons. To model the source and degree of differential item functioning, we used partial measurement invariance and dropped a discrimination item that did not reliably overlap with the latent factor for White women. After accounting for differential item functioning, Black women and men reported the highest discrimination, followed by Latinx women and men, and then White women and men. White women reported the most alcohol involvement, followed by White men, Latina women, Latino men, Black men, and Black women. Discrimination and alcohol involvement were positively associated for all groups except White women, though effect sizes varied with Black men exhibiting the largest effect. An intersectionally valid understanding of discrimination and alcohol involvement may necessitate statistical approaches that can test for (and model) differential item functioning prior to making between-group quantitative comparisons.

## Introduction

Alcohol use continues to be a substantial public health concern especially among young adults (ages 18–26) [1]. Indeed, over the past three decades the level of drinking has increased among young adults [2]. Data from Monitoring the Future shows that as many as 1 in 10 young adults are engaging in high-intensity drinking (e.g., consuming 10 or more drinks in a row) [3]. Thus, young adults are not merely experimenting with alcohol but engaging in drinking patterns with potential detrimental health effects. This is concerning given that there is continuity of drinking patterns into adulthood (at least for some) [4] coupled with a progressive increase in adult alcohol-related mortality [5] that has accelerated since the COVID-19 pandemic [6]. Thus, young adulthood represents a vulnerable developmental period when entrenched alcohol involvement patterns may evolve highlighting the need to understand risk factors of young adult alcohol involvement. Research suggests that experiences of discrimination (e.g., the mistreatment of individuals because of their membership in socially defined groups [7]) are linked to alcohol involvement [8]. Discrimination during young adulthood has been hypothesized to be a catalyst for both short-term and long-term negative health effects [9–12], and stress/coping frameworks have posited increased drinking as a behavioral determinant of the negative effects of discrimination [13].

However, despite the U.S. being an increasingly diverse society, there is a dearth of alcohol use research centering the role of demographic identity characteristics. For example, like academia and society at large [14], most mainstream alcohol research is “colorblind” and “genderblind.” That is, research typically either ignores race/ethnicity and gender variables or uses them as covariates to “wash away” or “control for” group similarities/differences [15–17]. Ignoring or controlling for effects of race/ethnicity and/or gender is a statistical limitation because if substantive group differences exist results will likely not generalize [18, 19]. Additionally, because of its socially constructed nature, using race/ethnicity as a predictor (main effect or covariate) in quantitative models has been criticized as being epistemically impossible because these models (e.g., regression) assume that predictors (e.g., race/ethnicity) cause the outcome variable (e.g., alcohol use), and hence using race/ethnicity as a predictor obscures our understanding of the processes that are likely to drive such associations [20]. Statistical approaches that quantify psychological aspects that vary across individuals have been argued to be a more informative approach [21, 22].

In efforts to elucidate variability due to overlapping race/ethnicity and gender identities, intersectionality as a methodological framework may be particularly useful [23–25]. From an intersectional theoretical perspective all people are characterized by multiple social identities that reflect dimensions of inequality and power [26–28], and hence covarying out potential race/ethnicity and/or gender differences functions as an impediment for understanding how overlapping identities relate to alcohol involvement [29, 30]. In other words, studies that estimate a main effect of race/ethnicity (i.e., a covariate) collapsing across gender, or that estimate a main effect of gender collapsing across race/ethnicity, are not methodologically able to ask if effects vary as a function of intersecting identities [31, 32]. As there is a need to better understand how social determinants can impact alcohol use across race/ethnicity and gender [33, 34] the present study examines the role of experiences of discrimination and young adult alcohol involvement at the intersection of race/ethnicity (Black, Latinx, and White) and gender (women and men).

Previous research has been conducted from a single-axis perspective (focusing on race/ethnicity but excluding gender or vice versa) and suggests that rates of drinking differ by race/ethnicity and gender. Studies have found that White adults report a higher prevalence of drinking than Black or Latino adults, with Black and Latino adults drinking comparably [3, 35, 36]. Historically men have been found to consume more alcohol than women though studies suggest the gender gap has decreased, closed, or even reversed [37–39] potentially due to changing gender norms [40, 41]. However, there is limited research on alcohol use patterns and associated risk factors at the intersection of race/ethnicity and gender which may hinder the generalizability of research findings.

Similarly, previous research has shown that experiences of discrimination are not equally distributed across groups [8, 42]. Research shows that Black participants score the highest on discrimination, White participants score the lowest and Latinx participants experience levels in between [43]. Furthermore, not only do participants who identify as Black report higher levels of perceived discrimination but also previous research has found the magnitude of the relationship between discrimination and substance use to be larger in Black samples [44]. Although, again, there is very little research at the intersection of race/ethnicity and gender which potentially limits generalizability.

Further complicating matters, there is a dearth of research testing if our measurement instruments “work the same” (or display comparable psychometric properties) across cultural groups. Importantly, valid between-group quantitative comparisons rest on the assumption of equivalent psychometric functioning of research tools across the groups being compared [45, 46]. Psychometric critiques of cross-cultural research document that most studies tend to make between-group inferences without testing if instruments work the same across the groups compared [47–49]. Psychometric critiques of cross-cultural research have existed for over three decades [50, 51]. Although Helms’ psychometric critique has received empirical support in recent years [52–54], most studies continue to neglect the possibility that between-group similarities/differences may be spuriously driven by differential item functioning. Hence, testing if research instruments work the same across groups is a necessary step before asking if there are between-group similarities/differences in discrimination and alcohol involvement among young adults.

There is a growing literature testing for the measurement invariance of alcohol and discrimination across race/ethnicity. These tests can identify bias in measurement and can attempt to model the degree of bias from a falsifiable framework [55]. Overall, studies have found that measures frequently display different psychometric properties across cultural groups yet the degree of bias can sometimes be modeled to allow for inferences at the latent level. Partial measurement invariance implies that, though observed/manifest scores may lead to misleading inferences, latent scores can correct for bias in measurement by precluding biased items from contributing equally to parameter estimates. For example, measures of alcohol use demonstrate differential item functioning across Black, Latinx, and White participants [56] yet the degree of measurement bias has been modeled (partial invariance) to allow for group comparisons at the latent level [35]; for similar conclusions about gender see Fish et al. [57]. Similarly, although Bastos and Harnois [58] found evidence for differential item functioning across Black, Latinx, and White participants using the Everyday Discrimination Scale; explicitly modeling such bias via partial invariance has allowed for group comparisons at the latent level [35, 59, 60]. However, there is a paucity of research testing the functioning of discrimination measurement across gender. In sum, when investigating the role of discrimination and alcohol use across the intersection of race/ethnicity and gender it may be important to test the psychometric comparability of research instruments prior to making group comparisons [61].

### The Present Study

**Fig. 1:**
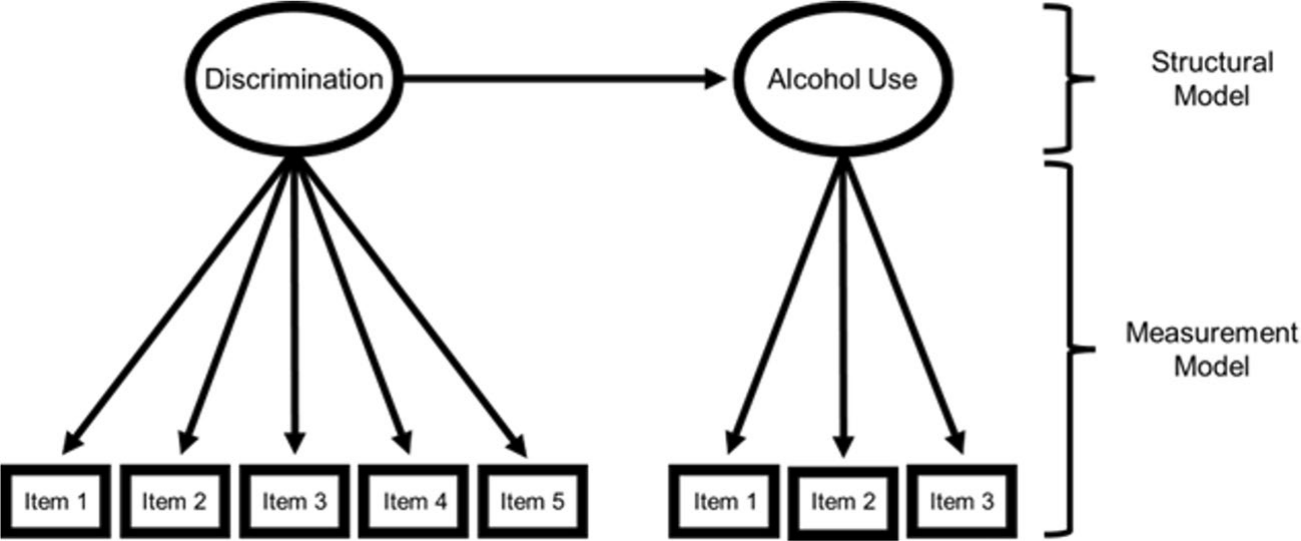
Visual depiction of structural equation model (SEM). Note: The measurement model estimates the internal consistency (reliability) of measurement; the structural model estimates the association between the latent variables

This study uses structural equation modeling (SEM) to investigate variability in experiences of discrimination and alcohol use. As shown in Fig. 1, SEM consists of two integrated sets of equations: (1) A measurement model that estimates the relationship of the observed indicators (e.g., questionnaire items) and the latent variable; and (2) a structural model that estimates the relationship between the latent factors. The measurement model provides an empirical estimate of reliability of measurement, whereas the structural model estimates the relationship between latent variables after accounting for measurement error. Specifically, we apply multigroup extensions of the SEM model shown in Fig. 1 that have been developed to ask if measures “work the same” (are equally reliable) across the groups being compared [62, 63]. To provide a valid quantitative test of between-group differences in dose of exposure to experiences of discrimination, we followed recommendations for testing for measurement invariance prior to making between-group inferences [49, 64]. In other words, inferences in the structural model will only be conducted given evidence of comparable psychometric functioning in the measurement model (Fig. 1). If evidence for bias in measurement is found (i.e., different psychometric functioning of the measurement model across groups), we will implement partial measurement invariance techniques to statistically accommodate (or model) for the degree of differential item functioning [65] (see Approach to Analyses section).

Although intersectionality facilitates a focus on overlapping identities, it emphasizes subjectivity and flexibility in what concurrent identities are investigated [28]. As tests of measurement invariance necessitate relatively large samples of individuals for every group investigated (~ 200 per group), and because of the well documented history of Black and Latinx oppression within the U.S. context [66], we framed this study on overlapping identities between the three largest racial/ethnic groups in the U.S. (Black, Latinx, and White) and binary cisgender (women and men). We consciously equated Latinx identity as its own category to be consistent with how many in such group identify themselves [67] as well as their shared history of oppression [68, 69]. Hence, we sampled young adults who identified as Black women, Latina women, White women, Black men, Latino men, and White men.

Finally, a note on operational definitions of cultural equivalence testing is warranted. This study focuses on testing the cultural equivalence of the measurement model shown in Fig. 1. From an SEM perspective, testing for group similarities/differences in the structural model necessitates psychometric equivalence in the measurement model (for a review see [49]). Although other forms of cultural equivalence testing exist (e.g., linguistic equivalence and content/construct validity), the focus on psychometric equivalence is necessary to examine intersectional variability in the association between discrimination and alcohol use. This is important because the idea of “switch intersectionality” [70] suggests that the etiology of constructs can show variability across intersectional identities, though tests of switch intersectionality have been neglected due to colorblind and genderblind practices in academia [14]. This study seeks to address these limitations of the alcohol use literature by testing the following questions: (1) Are there mean level differences in experiences of discrimination across intersectional race/ethnicity and gender identities? (2) Are there mean level differences in self-reported alcohol involvement across intersectional race/ethnicity and gender identities? (3) And does the degree of overlap between discrimination and alcohol involvement vary across intersectional race/ethnicity and gender identities?

## Methods

### Transparency and Openness

We acknowledge that the design and analysis plan for the study were not preregistered. The de-identified data and code files reported are publicly available and archived at the Open Science Framework (OSF; here). The research was approved by the institutional review board of the University of Rhode Island (protocol# IRB2021-139).

### Participants

The sample consisted of *n* = 1,187 emerging adults (mean age = 22.14, *SD* = 2.36). Specifically, there were *n* = 193 participants who identified as Black Women, *n* = 209 who identified as Latina Women, *n* = 186 White Women, *n* = 198 who identified as Black Men, *n* = 203 who identified as Latino Men, and *n* = 198 who identified as White Men. Sample demographic characteristics by race/ethnicity are presented in Table 1 for women and Table 2 for men.

**Table 1:** Sample demographic characteristics—women

	Total sample	Black women	Latina women	White women
*n* (%)	1187 (100%)	193 (16.26%)	209 (17.61%)	186 (15.67%)
Age—M (*SD*)	22.14 (2.36)	21.71 (2.42)	21.6 (2.35)	21.98 (2.34)
Orientation				
Heterosexual	900 (75.8%)	134 (69.43%)	134 (34.11%)	119 (63.98%)
Homosexual	48 (4%)	6 (3.11%)	6 (2.87%)	8 (4.3%)
Bi-sexual	213 (17.9%)	48 (24.87%)	60 (28.71%)	54 (29.03%)
Prefer not to say	8 (0.7%)	1 (0.52%)	3 (1.44%)	1 (0.54%)
Prefer to self-describe	18 (1.5%)	4 (2.07%)	6 (2.87%)	4 (2.15%)
Education level				
Less than high school	22 (1.9%)	3 (1.55%)	1 (0.48%)	2 (1.08%)
High school or equivalent	269 (22.7%)	41 (21.24%)	42 (20.1%)	20 (10.75%)
Some college	421 (35.5%)	69 (35.75%)	78 (37.32%)	70 (37.63%)
Associate’s	97 (8.2%)	9 (4.66%)	18 (8.61%)	12 (6.45%)
Bachelor’s	328 (27.6%)	59 (30.57%)	65 (31.1%)	74 (39.78%)
Other	50 (4.2%)	12 (6.22%)	5 (2.39%)	8 (4.3%)
College student				
Yes	578 (48.7%)	106 (54.92%)	123 (58.85%)	98 (52.69%)
No	609 (51.3%)	87 (45.08%)	86 (41.15%)	88 (47.31%)
Employment status				
Employed full-time	353 (29.7%)	48 (24.87%)	39 (18.66%)	61 (32.8%)
Employed part-time	186 (15.7%)	28 (14.51%)	37 (17.7%)	26 (13.98%)
Employed part-time (1–19 h)	134 (11.3%)	27 (13.99%)	26 (12.44%)	30 (16.13%)
Unemployed; looking for work	280 (23.6%)	48 (24.87%)	58 (27.75%)	23 (12.37%)
Unemployed; not looking for work	199 (16.8%)	34 (17.62%)	40 (19.14%)	44 (23.66%)
Homemaker	17 (1.4%)	5 (2.59%)	5 (2.39%)	2 (1.08%)
Unable to work	18 (1.5%)	3 (1.55%)	4 (1.91%)	0 (0%)
Current living situation				
With parents or other family member	610 (51.3%)	99 (51.3%)	121 (57.89%)	51 (27.42%)
With roommates on school campus	118 (9.9%)	27 (13.99%)	18 (8.61%)	30 (16.13%)
With roommates not on school campus	275 (23.2%)	31 (16.06%)	41 (19.62%)	78 (41.94%)
Living alone on school campus	35 (2.9%)	6 (3.11%)	5 (2.39%)	6 (3.23%)
Living alone not on school campus	135 (11.4%)	29 (15.03%)	21 (10.05%)	20 (10.75%)
Other	68 (5.7%)	1 (0.52%)	3 (1.44%)	1 (0.54%)
Income				
Under 15,000	565 (47.6%)	97 (50.26%)	120 (57.42%)	92 (49.46%)
16,000–30,000	203 (17.1%)	31 (16.06%)	36 (17.22%)	32 (17.2%)
31,000–45,000	172 (14.5%)	30 (15.54%)	28 (13.4%)	28 (15.05%)
46,000–60,000	116 (9.8%)	16 (8.29%)	14 (6.7%)	19 (10.22%)
61,000–75,000	59 (5%)	11 (5.7%)	8 (3.83%)	8 (4.3%)
76,000–90,000	37 (3.1%)	4 (2.07%)	1 (0.48%)	4 (2.15%)
Over 90,000	35 (2.9%)	4 (2.07%)	2 (0.96%)	3 (1.61%)
Family income				
Under 15,000	93 (7.8%)	14 (7.25%)	16 (7.66%)	4 (2.15%)
16,000–30,000	141 (11.9%)	25 (12.95%)	28 (13.4%)	9 (4.84%)
31,000–45,000	169 (14.2%)	26 (13.47%)	25 (11.96%)	13 (6.99%)
46,000–60,000	186 (15.7%)	34 (17.62%)	38 (18.18%)	25 (13.44%)
61,000–75,000	135 (11.4%)	16 (8.29%)	34 (16.27%)	20 (10.75%)
76,000–90,000	146 (12.3%)	31 (16.06%)	18 (8.61%)	31 (16.67%)
Over 90,000	315 (26.5%)	47 (24.35%)	49 (23.44%)	84 (45.16%)

**Table 2:** Sample demographic characteristics—men

	Total sample	Black men	Latino men	White men
*n* (%)	1187 (100%)	198 (16.68%)	203 (17.1%)	198 (16.68%)
Age—M (*SD*)	22.14 (2.36)	22.63 (2.21)	22.6 (2.38)	22.35 (2.25)
Orientation				
Heterosexual	900 (75.8%)	172 (86.87%)	173 (85.22%)	168 (84.85%)
Homosexual	48 (4%)	4 (2.02%)	13 (6.4%)	11 (5.54%)
Bi-sexual	213 (17.9%)	20 (10.10%)	16 (7.88%)	15 (7.58%)
Prefer not to say	8 (0.7%)	2 (1.01%)	0 (0%)	1 (0.51%)
Prefer to self-describe	18 (1.5%)	0 (0%)	1 (0.49%)	3 (1.52%)
Education level				
Less than high school	22 (1.9%)	3 (1.52%)	7 (3.45%)	6 (3.03%)
High school or equivalent	269 (22.7%)	77 (38.89%)	56 (27.59%)	33 (16.67%)
Some college	421 (35.5%)	63 (31.82%)	60 (29.56%)	81 (40.91%)
Associate’s	97 (8.2%)	13 (6.57%)	34 (16.75%)	11 (5.56%)
Bachelor’s	328 (27.6%)	36 (18.18%)	41 (20.20%)	53 (26.77%)
Other	50 (4.2%)	6 (3.03%)	5 (2.46%)	14 (7.07%)
College student				
Yes	578 (48.7%)	71 (35.86%)	96 (47.29%)	84 (42.42%)
No	609 (51.3%)	127 (64.14%)	107 (52.71%)	114 (57.58%)
Employment status				
Employed full-time	353 (29.7%)	70 (35.35%)	59 (29.06%)	76 (38.38%)
Employed part-time	186 (15.7%)	37 (18.69%)	32 (15.76%)	26 (13.13%)
Employed part-time (1–19 h)	134 (11.3%)	8 (4.04%)	18 (8.87%)	25 (12.63%)
Unemployed; looking for work	280 (23.6%)	58 (29.29%)	60 (29.56%)	33 (16.67%)
Unemployed; not looking for work	199 (16.8%)	21 (10.61%)	28 (13.79%)	32 (16.16%)
Homemaker	17 (1.4%)	3 (1.52%)	2 (0.99%)	0 (0%)
Unable to work	18 (1.5%)	1 (.051%)	4 (1.97%)	6 (3.03%)
Current living situation				
With parents or other family member	610 (51.3%)	114 (57.58%)	142 (69.95%)	83 (41.92%)
With roommates on school campus	118 (9.9%)	14 (7.07%)	9 (4.43%)	20 (10.10%)
With roommates not on school campus	275 (23.2%)	26 (13.13%)	31 (15.27%)	68 (34.34%)
Living alone on school campus	35 (2.9%)	8 (4.04%)	5 (2.46%)	5 (2.53%)
Living alone not on school campus	135 (11.4%)	34 (17.17%)	10 (4.93%)	21 (10.61%)
Other	68 (5.7%)	2 (1.01%)	6 (2.96%)	1 (0.51%)
Income				
Under 15,000	565 (47.6%)	71 (35.86%)	94 (46.31%)	91 (45.96%)
16,000–30,000	203 (17.1%)	38 (19.19%)	36 (17.73%)	30 (15.15%)
31,000–45,000	172 (14.5%)	26 (13.13%)	32 (15.76%)	28 (14.14%)
46,000–60,000	116 (9.8%)	28 (14.14%)	20 (9.85%)	19 (9.6%)
61,000–75,000	59 (5%)	10 (5.05%)	9 (4.43%)	13 (6.57%)
76,000–90,000	37 (3.1%)	11 (5.56%)	6 (2.96%)	11 (5.56%)
Over 90,000	35 (2.9%)	14 (7.07%)	6 (2.96%)	6 (3.03%)
Family income				
Under 15,000	93 (7.8%)	29 (14.65%)	20 (9.85%)	10 (5.05%)
16,000–30,000	141 (11.9%)	26 (13.13%)	41 (20.20%)	12 (6.06%)
31,000–45,000	169 (14.2%)	40 (20.20%)	45 (22.17%)	20 (10.10%)
46,000–60,000	186 (15.7%)	37 (18.69%)	37 (18.23%)	15 (7.58%)
61,000–75,000	135 (11.4%)	18 (9.09%)	19 (9.36%)	28 (14.14%)
76,000–90,000	146 (12.3%)	14 (7.07%)	17 (8.37%)	35 (17.68%)
Over 90,000	315 (26.5%)	34 (17.17%)	24 (11.82%)	77 (38.89%)

### Procedures

We contracted a leading company in conducting scientific-grade online platform research, X&Y Analytics (https://www.xandyanalytics.com/). Data collection was conducted as part of a lager study [71]. Inclusion criteria included being between 18 and 26 years of age and residing in the U.S. Recruitment was balanced on race/ethnicity (self-identifying as Black, Latinx/a/o, or White) and sex (self-describing biological sex assigned at birth). Once enrolled in the study gender identity was assessed (i.e., participants selected a gender identify from the following options: woman, man, non-binary, prefer to self-describe, and prefer not to say). The present analyses focus on cisgender identity (gender identity aligns with sex assigned at birth) [72]. Participants provided consent via an online form and thereafter completed a series of surveys split into two segments. The first segment was completed by 2100 participants. One-hundred and seventy-four cases were removed because they failed the attention check. An additional 101 cases were removed because their demographic responses did not align to the targeted demographics. A total of 1825 participants were invited to participate in segment two, of which 1375 participated. We conducted the same attention check and removed another 75 cases. Fifty-four cases were removed because their segment one and segment two data did not match. Based on the self-described demographic information 95% of the 1246 sample were cisgender. Given that conducting measurement invariance analyses requires large and about equal sample sizes in the groups being compared [73, 74], our final sample consisted of *n* = 1187 who identified as cisgender. Our focus on binary cisgender was due to budgetary limitations that precluded proactively recruiting a large enough sample that was diverse across race/ethnicity and other gender identities.

### Measures

Perceived discrimination was measured using five items from the Everyday Discrimination Scale [75, 76]. The items index various forms of mistreatment including: “being treated with less courtesy and respect,” “receiving poorer service at restaurants/stores”, “people acting as if you are not smart,” “people acting afraid of you,” and “being threatened or harassed.” Response options were frequency of occurrence ranging from “0 = never,” “1 = less than once per year,” “2 = a few times a year,” “3 = a few times a month,” “4 = at least once per week,” and “5 = almost every day.”

Individual differences in alcohol consumption were assessed using three items [77] including: (1) average frequency of alcohol use in the past six months on a Likert-scale (0 = “I have not used alcohol in the past 6 months,” 1 = “Less than 1 day a month,” 2 = “1 to 2 days a month,” 3 = “3 to 5 days a month,” 4 = “6 to 9 days a month,” 5 = “10 to 14 days a month,” 6 = “15 to 19 days a month,” 7 = “20 to 24 days a month,” 8 = “25 to 29 days a month,” and 9 = “All 30 days a month”); (2) average quantity of alcohol use per drinking occasion in the past 6 months ranging on a Likert-scale (0 = ”no use,” 1 = “Less than 1 full drink,” 2 = “1 or 2 drinks,” 3 = “3 or 4 drinks,” 4 = “5 or 6 drinks,” 5 = “7 or 8 drinks,” 6 = “9 or 10 drinks,” and 7 = “11 + drinks”); and (3) maximum number of standard drinks consumed in one drinking occasion in the past 6 months, asking respondents to give a number from “0–infinity.”

## Approach to Analyses

Multi-group SEM was applied using the software Mplus Version 8 [78]. Multi-group SEM is well suited for quantitative intersectionality research for the following reasons: it estimates unique parameter estimates for the six intersectional groups (i.e., does not have to rely on interactive race/ethnicity × gender variables); it allows for falsifiable tests of similarities/differences across these parameter estimates, and as a random effects model allows for within-group variability [79, 80]. We followed recommendations [63] to test for equivalent instrument functioning by examining three forms of measurement invariance: (1) configural invariance—or equivalent latent structure across groups; (2) metric invariance—or equality of item factor loadings across groups; and (3) scalar invariance—or equality of item intercepts across groups. Specifically, we utilized a series of confirmatory factor analyses (CFA) to test increasingly restrictive model specifications and examine if the fit of such nested models decreases [49]. For comparability with the literature, prior to testing for measurement invariance, a CFA on each measure was conducted without modeling race/ethnicity or gender (i.e., the color- and genderblind models).

When testing for measurement invariance, the first step tests for configural invariance by estimating a multi-group CFA model that allows factor loadings and item intercepts to differ across groups. Establishing configural invariance provides evidence that we are likely measuring the same construct across groups and is supported by results indicating that all groups have statistically significant (i.e., *p* < 0.05) and substantial factor loadings (i.e., standardized factor loadings > 0.3) as well as by an adequate baseline model fit. Adequate baseline model fit is evaluated by concurrently inspecting distinct model fit metrics, typically considered comparative model fit (*CFI*) values > 0.90, root mean square error of approximation (*RMSEA*) values < 0.08, and standardized root mean squared residual (*SRMR*) values < 0.08.

Step 2, metric invariance, involves constraining factor loadings to be equal across groups and testing for decrements in model fit. Metric invariance provides evidence that the overlap between the items and the latent construct is comparable across groups and is a statistical assumption needed to compare covariances across groups [81]. Scalar invariance (step 3) involves constraining item intercepts to be equal and testing for decrements in model fit. Scalar invariance provides evidence that change in the latent variable leads to a comparable degree of changes in the manifest variables across groups and is a statistical assumption needed to compare means across groups [82]. We follow recommendations to concurrently consider decrements in multiple model fit metrics [63]. Specifically, decrements in *CFI* > 0.01 are considered substantial, decrements in *RMSEA* > 0.015 are considered substantial, and decrements in *SRMR* > 0.03 are considered substantial [83, 84].

Bias in measurement is not an all-or-none outcome; if some degree of measurement bias is detected, it is possible to attempt to model such differences in item functioning [85]. Upon detection of differential item functioning, parameters that vary across groups were unconstrained and decrements in model fit examined [49, 62]. This approach uses latent scores to correct for bias in measurement by precluding biased items from contributing equally to parameter estimates. After testing for differential instrument functioning across race/ethnicity and gender, latent mean and covariance differences were tested. Finally, we estimated the statistical significance of within-group variability in parameter estimates (i.e., random effects).

## Results

### Measurement Invariance for Experiences of Discrimination

Collapsing across race/ethnicity and gender a CFA with one factor suggested a strong overlap between the items (*β’*s range from 0.57 to 0.82, all *p-values* < 0.01), which fit the data well as indicated by *CFI* = 0.982, *RMSEA* = 0.073, and *SRMR* = 0.022. Although a multigroup *CFA* fit the data well (see Table 3), and all factor loadings were statistically significant, the item “being threatened or harassed” had a non-substantial factor loading for White women. The standardized factor loading for White women was *β* = 0.25, indicating only 6% of the item variance can be predicted by the latent variable (for Black women *β* = 0.61, Latina women *β* = 0.39, Black men *β* = 0.63, Latino men *β* = 0.71, and White men *β* = 0.74). Facing the option of either removing White women from the analyses or dropping the item, we opted to drop this item from further analyses for all groups.

**Table 3:** Summary of tests of measurement invariance

		*CFI*	*RMSEA* (90% *CI*)	*SRMR*	Model comparison	Δ*CFI*	Δ*RMSEA*	Δ*SRMR*	Decision
*Experiences of discrimination*									
Model 1	Configural invariance	.942	.111(.092–.131)	.068	–-	–-	–-	–-	Reject
Model 1b	Configural invariance (dropping item 5)	.962	.114 (.086–.142)	.054	–-	–-	–-	–-	Accept
Model 2	Metric invariance	.929	.112 (.092–.132)	.129	Model 1b	.033	.002	.075	Reject
Model 2b	Partial metric invariance	.954	.113 (.088–.138)	.068	Model 1b	.008	.001	.014	Accept
Model 3	Scalar invariance	.954	.113 (.088–.138)	.068	Model 2b	0	0	0	Accept
*Alcohol Involvement*									
Model 1	Configural invariance	.989	.058 (.000–.106)	.026	–-	–-	–-	–-	Accept
Model 2	Metric invariance	.930	.093 (.067–.120)	.151	Model 1	.059	.035	.125	Reject
Model 2b	Partial metric invariance	.991	.038 (.000–.077)	.041	Model 1	0	0	.015	Accept
Model 3	Scalar invariance	.991	.038 (.000–.077)	.041	Model 2b	0	0	0	Accept

Discrimination model 1 was rejected because discrimination item number 5 had a standardized factor loading of .25 in White women; discrimination model 2b has unconstrained factor loadings for discrimination items 1–3; for alcohol model 2b, factor loadings were constrained for all women separately from men; factor loadings were constrained for Black and Latinx men but unconstrained for White men

*CFI* Comparative Fit Index, *RMSEA* root mean square error of approximation, *SRMR* standardized root mean square residual, *CI* confidence interval, *Decision “Accept”* accepting the assumptions of invariance, *decision “Reject”* rejecting the assumptions of invariance

As shown in Table 3, a configural invariance model (dropping the item “being threatened or harassed”) fit the data well, though *RMSEA* was right outside of conventional boundaries of adequate model fit (*RMSEA* = 0.11). As *CFI* and *SRMR* have been shown to be less susceptible to model complexity than *RMSEA* [86], and *RMSEA* is right outside of an arbitrary boundary [87, 88], we argue that, overall, there is sufficient evidence of adequate model fit to establish configural invariance. All factor loadings were statistically significant (*p* < 0.05) and substantial (*β* > 0.30) (see Table 4).

**Table 4:** Standardized factor loadings for discrimination factor

	Black women	Latina women	White women	Black men	Latino men	White men
1. Less courtesy/respect	*β* = .79*	*β* = .67*	*β* = .49*	*β* = .75*	*β* = .84*	*β* = .93*
2. Poorer services restaurants stores	*β* = .76*	*β* = .79*	*β* = .61*	*β* = .73*	*β* = .62*	*β* = .66*
3. People act as if you are not smart	*β* = .69*	*β* = .46*	*β* = .31*	*β* = .66*	*β* = .80*	*β* = .79*
4. People act afraid of you	*β* = .56*	*β* = .58*	*β* = .56*	*β* = .73*	*β* = .47*	*β* = .63*

Discrimination item number 5 was dropped due to a non-substantial factor loading (.25) in White women; residuals for items 1 and 3 were allowed to covary due to modification indices

^*^ *p* < .05

As shown in Table 3, constraining item factor loadings (i.e., metric invariance) to be equal across groups resulted in substantial decrements in model fit. Hence, the assumption of metric invariance was rejected. To obtain partial metric invariance the factor loadings for “less courtesy/respect,” “poorer services at restaurants or stores,” and “people acting as if you are not smart” were unconstrained. As shown in Table 3, this partial metric invariance model fit the data as well as the configural invariance model which supports the assumption of partial metric invariance. Finally, constraining item intercepts across groups led to no decrements in model fit as indicated by *CFI*, *RMSEA,* and *SRMR* (see Table 3), providing evidence for the assumption of scalar invariance.

### Measurement Invariance for Alcohol Involvement

Collapsing across race/ethnicity and gender, a *CFA* with one factor suggested a strong overlap between the past 6-month frequency of drinking (*β* = 0.82, *p* < 0.01), average quantity of drinking (*β* = 0.90, *p* < 0.01), and maximum drinks on one occasion (*β* = 0.87, *p* < 0.01). Although model fit cannot be estimated with only three indicators in a mono-group *CFA*, a multi-group *CFA* was conducted and yielded adequate fit to the data (see Table 3). All factor loadings for the alcohol use items were statistically significant (*p* < 0.05) and substantial with standardized factor loadings ranging from *β* = 0.73 to *β* = 0.95.

As shown in Table 3, constraining item factor loadings to be equal across groups resulted in substantial decrements in model fit. Hence, the assumption of metric invariance was rejected. To obtain partial metric invariance, factor loadings were constrained for all women separately from men and separately for white men but constrained for Black and Latinx men. Although standardized parameters differ across groups due to distinct distributions, for women the unstandardized factor loadings were as follows: frequency *b* = 1.398 (*p* < 0.01), quantity *b* = 1.189 (*p* < 0.01), max drink *b* = 2.883 (*p* < 0.01). For Black and Latino men: frequency *b* = 1.870 (*p* < 0.01), quantity *b* = 1.675 (*p* < 0.01), and max drink *b* = 3.454 (*p* < 0.01); whereas for White men: frequency *b* = 1.754 (*p* < 0.01), quantity *b* = 1.458 (*p* < 0.01), and max drink *b* = 4.455 (*p* < 0.01). These correspond to standardized factor loadings for women between *β* = 0.72 and 0.79 for frequency, between *β* = 0.82 and 0.88 for quantity, and between *β* = 0.85 and 0.96 for max drink. For Latino and Black men, standardized factor loadings range between *β* = 0.86 and 0.87 for frequency, between *β* = 0.94 and 0.96 for quantity, and between *β* = 0.82 and 0.87 for max drink. For White men standardized factor loadings were: *β* = 0.79 for frequency, *β* = 0.86 for quantity, and *β* = 0.87 for max drink. As shown in Table 3, the partial metric invariance model fit the data as well as the configural invariance model which supports the assumption of partial metric invariance. Finally, constraining item intercepts across groups yielded the same model fit as the partial metric invariance model providing evidence for the assumption of scalar invariance (Table 3).

### Latent Mean Differences

Latent mean differences across groups were estimated using the final scalar invariance models, setting the latent mean for black women to equal zero, freely estimating the latent mean for all other groups, and testing if such means are statistically significantly different from other group means. We note that the latent mean coefficients are standardized and can be interpreted in standard deviation units (an effect size estimate). Using common guidelines [89, 90] coefficients in the range of 0.10, 0.30, and 0.50 indicate small, medium, and large effects (respectively).

**Fig. 2:**
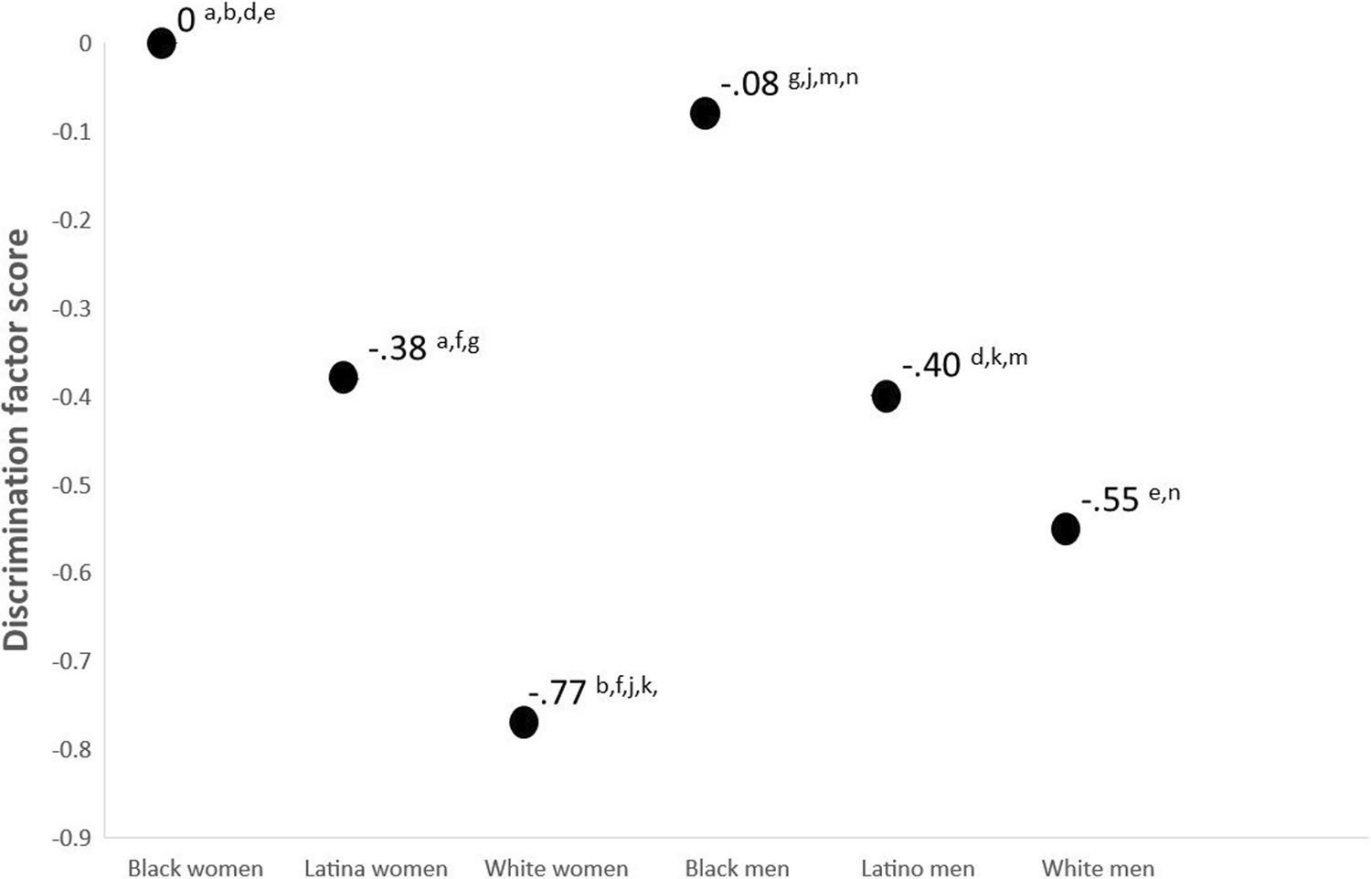
Latent mean differences for experiences of discrimination factor. Notes: Subscripts refer to statistically significant differences between the following mean contrasts: a Black women–Latina women; b = Black women–White women, c = Black women–Black men; d = Black women–Latino men; e = Black women–White men; f = Latina women–White women; g = Latina women–Black men; h = Latina women–Latino men; i = Latina women–White men; j = White women–Black men; k = White women–Latino men; l = White women–white men; m = Black men–Latino men; *n* = Black men–White men; o = Latino men–White men

Figure 2 shows the latent means for experiences of discrimination and subscripts refer to statistically significant mean contrasts. Black women scored the highest in the discrimination factor, although their factor score was not statistically significantly different from Black men (who scored 0.08 standard deviation units less than Black women). Tests of mean contrasts suggest that the mean of discrimination for Black women was statistically significantly different than the mean for Latina women, White women, Latino men, and White men. Similarly, the mean for Black men was statistically significantly different from the means of Latina women, White women, Latino men, and White men. Overall, White women reported the lowest levels of discrimination, and their mean was 0.77 standard deviation units lower compared to Black women. Tests of random effects suggest that there is a substantial amount of within-group variability in experiences of discrimination for all groups (all *p*’s < 0.01).

**Fig. 3:**
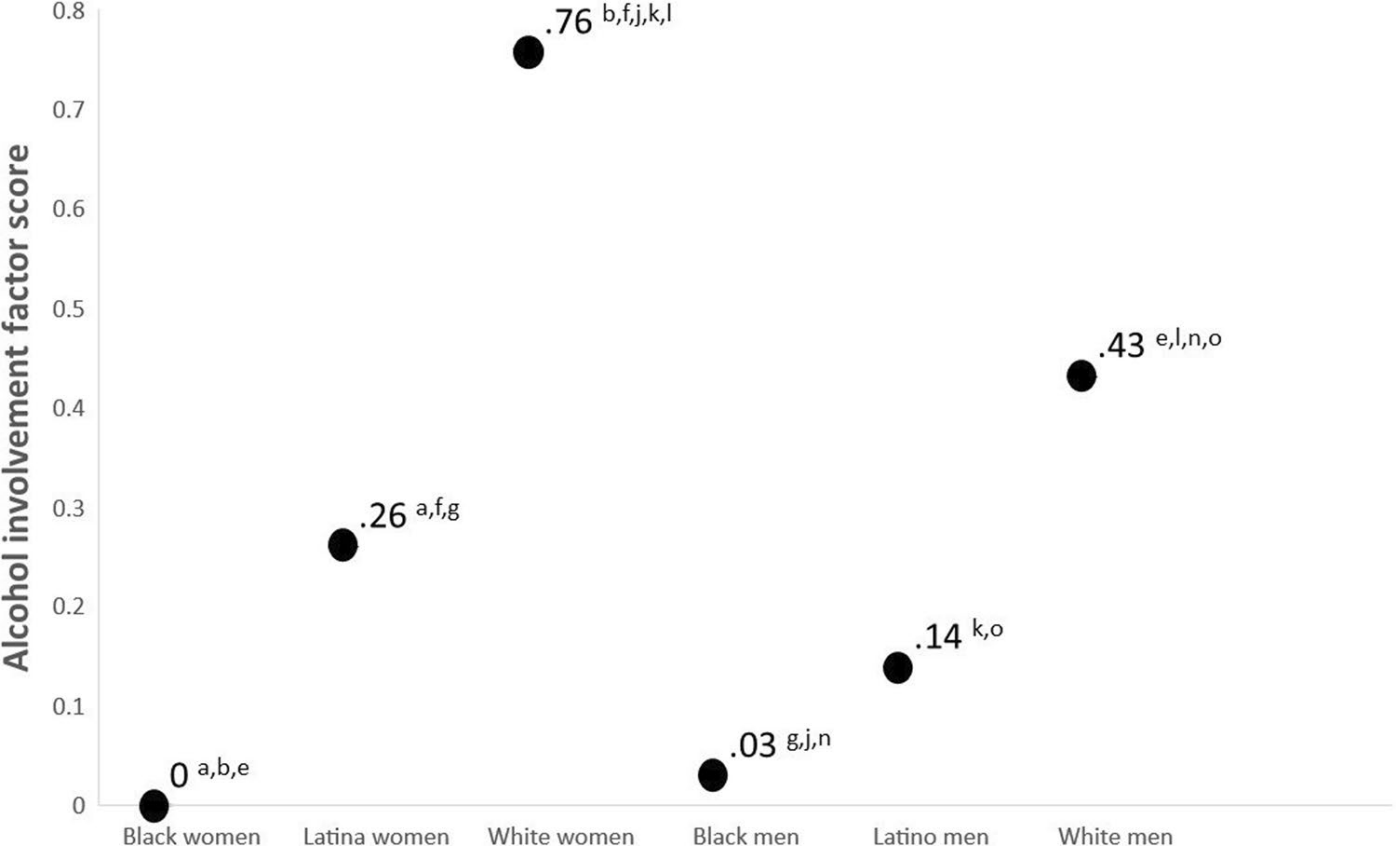
Latent mean differences for alcohol involvement factor. Notes: Subscripts refer to statistically significant (*p* < .05) differences between the following mean contrasts: a Black women–Latina women; b Black women–White women, c Black women–Black men; d Black women–Latino men; e Black women–White men; f = Latina women–White women; g = Latina women–Black men; h = Latina women–Latino men; i = Latina women–White men; j = White women–Black men; k = White women–Latino men; l = White women–white men; m = Black men–Latino men; n = Black men–White men; o = Latino men–White men

Figure 3 displays the latent means for alcohol use and subscripts refer to statistically significant mean contrasts. White women scored the highest in the alcohol factor and mean contrast tests suggest that their score is statistically significantly higher than the mean for all other groups. Black women scored the lowest in the alcohol involvement factor, although their score was not statistically significantly different than that of Black men who scored 0.03 standard deviations higher. Random effects suggests that there is a substantial amount of within-group variability in alcohol use for all groups (all *p*’s < 0.01).

### Latent Alcohol Use Regressed on Latent Discrimination

**Fig. 4:**
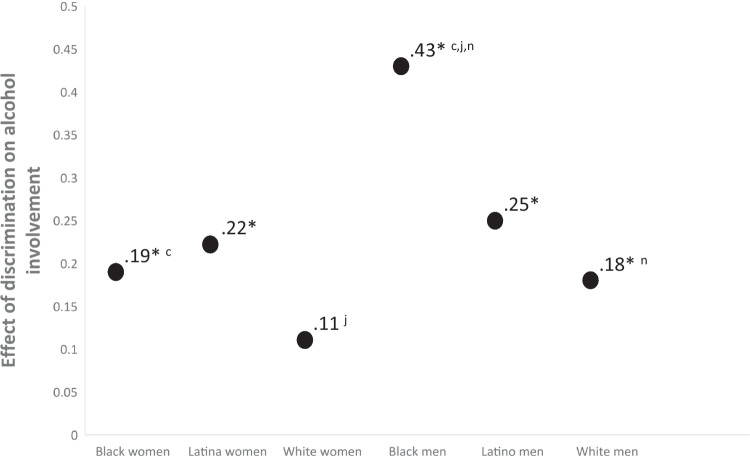
Differences in effect sizes of the overlap between discrimination and alcohol involvement. Notes: * = *β* significantly different from zero (*p* < .05); subscripts refer to statistically significant (*p* < .05) differences between the following *β* contrasts: a = Black – Latina women; b = Black women–White women; c = Black women–Black men; d = Black women–Latino men; e = Black women–White men; f = Latina women–White women; g = Latina–Black men; h = Latina women–Latino men; i = Latina women–White men; j = White women–Black men; k = White women–Latino men; l = White women–white men; m = Black men–Latino men; n = Black men–White men; o = Latino men–White men

Figure 4 shows the effect sizes of the overlap between variability in experiences of discrimination and alcohol involvement. More experiences of discrimination predicted more alcohol involvement for all groups except White women, though the trend for White women was in the same direction (*β* = 0.11, *p* = 0.34). Effect sizes for all other groups were statistically significant with effect sizes between small and medium, except for Black men. For Black men a one-standard deviation unit change in discrimination was associated with a 0.43 standard deviation unit change in alcohol use, which is indicative of a medium to large effect size.

### Test of Alternative Models

To empirically explore the ramifications of opting to drop the discrimination item “being threatened or harassed,” we re-ran our analyses excluding White women (for whom the item showed inadequate reliability) but including the item. These alternative five-group models showed comparable inferences. Hence, we retain the approach that includes White women. These alternative analyses are provided in the online OSF files (for the syntax and supplemental results see Table S1 https://osf.io/vjh26/?view_only=367a755a4c134b61b03f140297104a86).

## Discussion

Discrimination is an important social determinant of alcohol involvement and is particularly important during young adulthood when alcohol use peaks and life-long patterns of use are cemented [91]. However, most research has been conducted in predominantly White and genderblind samples, which has been hypothesized to be manifestation of structural racism and an obstacle for generalizability and innovation [15]. Below we discuss the alcohol and discrimination findings, psychometric findings, and the implications and future directions.

This study suggests that there is a large degree of variability in alcohol use across intersectional race/ethnicity and gender identities, variability that is typically “swept under the rug” by colorblind and genderblind research practices. Although our findings are consistent with research suggesting White young adults consume more alcohol than minoritized young adults [92], our intersectionally-minded methods elucidate that White women reported the most alcohol use in this sample (see Fig. 3). Specifically, White women reported drinking more than the second highest drinking group (White men) by 0.33 standard deviation units and reported drinking more than the lowest drinking group (Black women) by 0.76 standard deviation units. The higher rates of alcohol consumption in White young adults have been conceptualized to be the result of cultural differences in the acceptability of alcohol use in young adulthood [93]. Future longitudinal research is needed because there is evidence that risk for alcohol use among Black men increases in middle adulthood, potentially due to cumulative exposures to racism [94]. The high rates of discrimination among Black participants shown in Fig. 2 provide a cross-sectional snapshot of such exposure. Our data suggest that longitudinal studies may need to test these models across gender because Black women experienced comparable levels of discrimination to Black men.

Although historically men have reported more drinking than women, in this sample there were no gender differences in alcohol use among Black and Latinx young adults and large gender difference among White young adults. As women are less likely to access treatment than men [95], it may be important for public health to understand the changing landscape of alcohol consumption. Gender differences may be driven by changes in how people think about drinking (e.g., alcohol outcomes expectancies and motivations for use) as well as changes in traditional gender norms [96–98]. The results of this study suggest that future studies “unpacking” mechanisms driving gender differences in alcohol use may benefit from exploring variability across intersectional racial/ethnic identities.

Similarly, experiences of discrimination displayed a spectrum of exposure across intersecting race/ethnicity and gender identities. Although everyone reported experiences of interpersonal discrimination (poorer service at restaurants/stores, people acting afraid of you, etc.; see Fig. 2) Black young adults reported the highest levels followed by Latinx and White young adults. The results suggest that more discrimination is associated with more drinking for all groups except White women. Although the effect was not statistically significant for White women, the directionality of the effect is consistent with the other effects (i.e., a one-standard deviation unit increase in discrimination is associated with a 0.11 standard deviation unit increase in alcohol use among White women), which may be indicative of a small effect that may have been statistically significant with a larger sample size. Effects for all other groups were in the small to medium range except for a large effect for Black young adult men. In Black men 18% of the variance observed in drinking could be explained by discrimination. Although results from the current study cannot elucidate why the effect size of discrimination and drinking is larger in Black men, it is possible that the history of oppression among Black men in our society makes experiences of discrimination more impactful. These data suggest that the nomological network of alcohol involvement might show cultural variability at the intersection of race/ethnicity and gender. This finding is consistent with the concept of switch intersectionality or with the idea that a variable’s associative network can differ across intersectional identities. Future longitudinal studies are needed to inform an intersectionally valid understanding of the development of alcohol use.

### Psychometric Findings

This study demonstrates that quantitative intersectionality research likely necessitates a measurement framework that can test for differential item functioning. Quantitative models that omit tests of measurement invariance assume equivalent psychometric functioning [99]. In other words, manifest level tests of between-group similarities/differences are a special application of latent variable models, a special application that assumes that instruments display equal psychometric properties across groups [100, 101].

Most of the psychometric evaluations of the Everyday Discrimination Scale have occurred in predominantly Black samples [61], and the current study contributes to the literature by testing the psychometric comparability of the tool across race/ethnicity and gender among young adults. For experiences of discrimination, one item was found to function differently in White women. The item “being threatened or harassed” fell below conventional standards for reliability in White women, and because we had five variables (and latent variable modeling only necessitates three indicators per factor), we dropped the item from further analyses for all groups. We believe that our approach is valuable because measurement invariance is a necessary assumption for making group comparisons which can “map” differential exposures to social determinants of health. However, it is important to note that it is not theoretically necessary for constructs to have the same latent structure across groups [102]. To examine the impact of dropping this item, we conducted alternative analyses in which we kept the item but dropped White women from the analyses. These alternative analyses yield comparable inferences and are presented in the online OSF files.

Although we did not have a priori expectations that this item would not be reliable in White women, we offer possible post hoc explanations. The marginalization of White women in the U.S. has been postulated to be unique in that they are simultaneously both an oppressed group and a powerful group to be protected for the maintenance of White privilege [103, 104]. Hence, “threatening and harassing” White young adult women may be less frequent than in other intersectional identities in our society. This item is anchored on the word “threaten” and thus may have primed violent content which may be less common among White young women in the U.S. We acknowledge this is a post hoc explanation, though it does align with other findings [105]. However, it counters research showing that non-violent sexual harassment is common among women, including White women [106, 107]. Thus, another explanation for our finding may be that this item is not reliable for White women and a different item wording may be necessary for the item to be useful among White women. Lastly, these results may indicate that the five items included in this study do not represent a robust measure of discrimination and should not be utilized for between-group comparisons in samples that include White young adult women without first testing for differential item functioning. As psychometric properties are sample dependent [108, 109], these results suggest that future studies may benefit from explicitly considering differential item functioning. Studies that are not statistically powered to test for measurement invariance should discuss/contemplate the possibility that results are driven by (untested) differential item functioning.

The metric and partial metric invariance models suggest the remaining four discrimination items, although all “reliable enough,” differ in reliability across the groups sampled. For example, the factor loadings for the item “poorer service at restaurants or stores” could not be modeled to be equal across groups (i.e., the assumption of metric invariance was rejected), yet the difference in factor loadings in the partial metric invariance models had a range from *β* = 0.64 to 0.77, a maximum difference of 0.13 standard deviation units. In other words, all values in that range would be considered “reliable enough,” yet there are small differences in how reliable items are across groups. These differences violate assumptions of manifest-level between-group comparisons, specifically the assumption that all items are equally reliable across groups [49]. Violations of statistical assumptions decrease confidence in the validity and generalizability of findings. Latent variable models, such as partial invariance models, do not need to assume that items have the same degree of reliability across groups and hence may be well suited for testing similarities/differences across race/ethnicity and gender [85].

Alcohol measurement invariance models also suggested that although all items are “reliable enough,” between-group variability exists in how reliable they are. The partial metric invariance model for alcohol suggest that items differ in reliability across groups that ranges from *β* = 0.72 to 0.96, a maximum difference of 0.24 standard deviation units. These results provide evidence for psychometric critiques and suggest that structural equation modeling may be a useful tool for quantifying intersectional similarities/differences in alcohol use.

### Implications and Future Directions

This study addresses colorblind and genderblind limitations of the alcohol literature and suggest that research conducted on predominantly White and genderblind samples will likely make a large portion of the population scientifically invisible [110]. For example, almost 1/5 of the variance in drinking among Black men could be explained by experiences of discrimination, yet most the literature explains drinking as a function of individual-level mechanisms without contextualizing how environmental stressors influence outcomes [111]. Similarly, if this study had tested gender differences collapsing across race/ethnicity, the level of drinking for women would have been composed of the average for the groups (see Fig. 3). Although the outcomes of such aggregation would be dependent on relative sample sizes, a single-axis perspective would fail to see that White women reported more drinking than all men and Black and Latina women in this sample. If previous genderblind research on the within-person continuity of drinking into later developmental periods generalizes [4] our data suggests that the high levels of drinking found in White women may become a significant public health concern as young adults enter middle and older adulthood. Hence, there is a need for future research to move beyond current colorblind and genderblind practices.

It is well documented that most public health and traditional Western academic models usually place the onus of the problem within the person and ignores or minimizes social determinants of health [112, 113]. Our study suggests that theoretical models of alcohol that deemphasize social determinants may have limits to generalizability. A better understanding of alcohol use patterns as young adults learn to relate to alcohol can inform preventive and intervention efforts that are tailored to the individual. For example, interventions that frame alcohol use as an inadvertently self-defeating coping reaction to discrimination may energize coping strategies that are antagonistic to drinking. It has been speculated that the path towards clinical innovations may need to “map” alcohol related heterogeneity in within-person phenotypic traits [114] and contextual determinants of health [115], and our study suggests that these efforts may benefit from testing theoretical models of drinking across intersectional identities.

### Limitations

We contextualize these findings within the limitations of this study. Although “unpacking” effects across the three largest racial/ethnic groups in the U.S. and binary gender is an improvement over the majority of alcohol use research that collapses across these variables, these are only a few examples within a variety of intersectional identities. We note that we did not intend to only focus on cisgender individuals but did so due to 95% of the sample identifying as cisgender. Our statistical analyses required our groups to be of about equal size. Thus, future research with a focus on gender diversity may benefit from prioritizing gender identity as a study inclusion criterion. Furthermore, we highlight that many other intersectional identities (e.g., age, sexual orientation, non-binary gender, socioeconomic status, and religiosity) are likely important to examine for understanding the relationship between discrimination and alcohol involvement. Next, we acknowledge that our conceptualization of Latinx as its own racial/ethnic category has been critiqued as obscuring higher rates of discrimination among Afro-Latinos [116, 117]. Although there is no consensus on how to best conceptualize the Latino/a experience [118], future studies may benefit from further quantifying within-group heterogeneity. Similarly, our measure of experiences of discrimination may oversimplify the variety of discrimination experienced by individuals, and future studies may benefit from including other components of discrimination such as systemic racism [119]. Finally, directionality of effects is uncertain with cross-sectional data. Prospective studies are needed to ascertain how cumulative exposure to discrimination impacts alcohol across the lifespan.

## Conclusions

In sum, our results suggest that colorblind and genderblind models/methods may omit a substantial amount of heterogeneity in alcohol use. Although traditionally not included in public health models, experiences of discrimination predicted a non-trivial amount of variance in alcohol use, with almost 1/5 of the variance in drinking being predicted by discrimination in Black men. Although the findings of the current study cannot directly inform public health interventions, they do suggest that academic dialogue should focus on “unpacking” how social determinants of alcohol unfold across intersectional race/ethnicity and gender identities.

In an increasingly diverse society improving the public health of the nation may necessitate “unpacking” theoretical models across intersectional positions and such efforts may benefit from asking if our tools show evidence of comparable psychometric functioning across the groups being investigated.

## Data Availability

Data and syntax for this project are made available via the Open Science Framework: https://osf.io/vjh26/?view_only=367a755a4c134b61b03f140297104a86.
